# A comparative methylome analysis reveals conservation and divergence of DNA methylation patterns and functions in vertebrates

**DOI:** 10.1186/s12915-022-01270-x

**Published:** 2022-03-23

**Authors:** Hala Al Adhami, Anaïs Flore Bardet, Michael Dumas, Elouan Cleroux, Sylvain Guibert, Patricia Fauque, Hervé Acloque, Michael Weber

**Affiliations:** 1 University of Strasbourg, Strasbourg, France; 2 CNRS UMR7242, Biotechnology and Cell Signaling, 300 Bd Sébastien Brant, 67412 Illkirch Cedex, France; 3 Université Bourgogne Franche-Comté, Equipe Génétique des Anomalies du Développement (GAD) INSERM UMR1231, 2 Rue Angélique Ducoudray, 21000 Dijon, France; 4 CHU Dijon Bourgogne, Laboratoire de Biologie de la Reproduction – CECOS, 14 rue Gaffarel, 21000 Dijon, France; 5 Université Paris-Saclay, INRAE, AgroParisTech, GABI, 78350 Jouy-en-Josas, France

**Keywords:** DNA methylation, 5mC, Vertebrates, CpG island, Germline genes, Genomic imprinting

## Abstract

**Background:**

Cytosine DNA methylation is a heritable epigenetic mark present in most eukaryotic groups. While the patterns and functions of DNA methylation have been extensively studied in mouse and human, their conservation in other vertebrates remains poorly explored. In this study, we interrogated the distribution and function of DNA methylation in primary fibroblasts of seven vertebrate species including bio-medical models and livestock species (human, mouse, rabbit, dog, cow, pig, and chicken).

**Results:**

Our data highlight both divergence and conservation of DNA methylation patterns and functions. We show that the chicken genome is hypomethylated compared to other vertebrates. Furthermore, compared to mouse, other species show a higher frequency of methylation of CpG-rich DNA. We reveal the conservation of large unmethylated valleys and patterns of DNA methylation associated with X-chromosome inactivation through vertebrate evolution and make predictions of conserved sets of imprinted genes across mammals. Finally, using chemical inhibition of DNA methylation, we show that the silencing of germline genes and endogenous retroviruses (ERVs) are conserved functions of DNA methylation in vertebrates.

**Conclusions:**

Our data highlight conserved properties of DNA methylation in vertebrate genomes but at the same time point to differences between mouse and other vertebrate species.

**Supplementary Information:**

The online version contains supplementary material available at 10.1186/s12915-022-01270-x.

## Background

5-methylcytosine (5mC) is a key epigenetic modification known to be involved in biological processes such as regulation of gene expression, DNA structure and control of transposable elements. 5mC exists in most eukaryotic groups including plants, fungi, invertebrate and vertebrate animals [1]. It is however absent in certain model organisms such as the budding yeast *Saccharomyces cerevisiae*, the nematode worm *Caenorhabditis elegans* and the fly *Drosophila melanogaster*. Furthermore, the levels and genomic patterns of 5mC are evolutionarily labile. While invertebrate genomes display sparse methylation with most methylation accumulating in transcribed genes, vertebrate genomes are extensively methylated [2].

In vertebrate genomes, 5mC occurs predominantly in a CpG sequence context [3]. 5mC can be converted to thymine by spontaneous or enzymatic deamination, which is thought to lead to an evolutionary depletion of CpGs in methylated vertebrate genomes [4], except at CpG-rich regions known as CpG islands (CGIs) that remain mostly unmethylated in somatic cells and the germline [5].

In the well-studied mouse and human genomes, DNA methylation silences transposable elements and prevents them from disturbing expression of neighboring genes [6, 7]. In the mouse, CGI methylation is infrequent and occurs mostly in gene bodies [8]. At transcription start sites (TSS), most CGIs remain constitutively unmethylated, except a minor fraction undergoing long term silencing by DNA methylation associated with X-chromosome inactivation (XCI), parental genomic imprinting and developmental genes. In particular, promoter CGI DNA methylation is targeted to a small number of germline genes during mouse development and required to maintain these genes repressed in somatic lineages [6, 8, 9]. More recently, a new class of large unmethylated regions covering developmental genes, termed DNA methylation valleys or canyons, has been described in mouse, human and zebrafish [10–12].

While the current knowledge of mammalian DNA methylation largely stems from studies in mouse and human, little is known about the degree of conservation of DNA methylation distribution and functions in vertebrates. Some comparative studies addressed the evolutionary conservation of DNA methylation between plants, invertebrates and vertebrates [1, 13]. In vertebrates, comparative methylome studies were performed in sperm of mammals [14, 15] and organs of three primates [16]. It was also shown by purification of non-methylated DNA that unmethylated islands are a conserved feature of gene promoters in several vertebrates [17]. More recently, a study showed that CpH methylation occurs in the brain of all vertebrates [18]. To our knowledge, previous studies did not address the conserved functions of DNA methylation in vertebrates by DNA methylation inhibition.

We therefore wished to study the conservation of DNA methylation patterns by generating single-base methylation profiles in primary cells from six placental mammals and one bird. We show that, while the basic principles of the distribution of cytosine methylation are conserved across these species, the chicken genome is hypomethylated compared to mammals and the threshold of CpG density associated with protection from DNA methylation varies among species. We analyze the evolutionary conservation of DNA methylation patterns associated with developmental genes, X-chromosome inactivation and parental genomic imprinting. In addition, we interrogate the functions of DNA methylation in vertebrate cells by DNA methylation inhibition. Our data highlight both conservation and divergence in the distribution and functions of DNA methylation in vertebrates.

## Results

### Single-base methylomes in primary fibroblasts from seven vertebrates

**Fig. 1:**
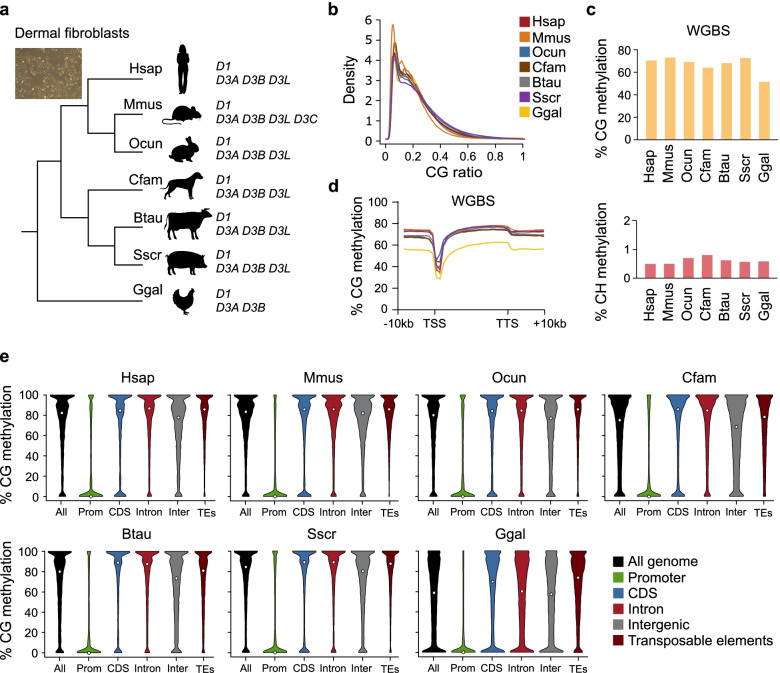
Single-base methylomes in primary dermal fibroblasts of seven vertebrate species. **a** Phylogenetic relationships between the studied species. For each species, we indicate whether the genome contains an annotated *DNMT1* (*D1*), *DNMT3A* (*D3A*), *DNMT3B* (*D3B*) and *DNMT3L* (*D3L*) gene. **b** Density plot of the distribution of CG ratios (CpG observed/expected) calculated in 0.5 kb genomic sliding windows. **c** Average methylation of CG (upper panel) and CH (lower panel) sites with at least 5 unique reads in WGBS datasets generated from dermal fibroblasts. **d** Metaplot of CG methylation levels over Ensembl genes and 10 kb flanking sequences calculated from the WGBS datasets in dermal fibroblasts. TSS: Transcription start site, TTS: Transcription termination site. **e** Violin plots of WGBS CG methylation levels in different genomic features in dermal fibroblasts. Median values are indicated by white circles

To study the conservation of DNA methylation patterns among vertebrates, we isolated genomic DNA from primary dermal fibroblasts from seven vertebrate species (human, mouse, rabbit, dog, cow, pig and chicken, Fig. 1a). In each species, we used primary fibroblasts that were cultured at very low passage (< P6) following derivation and were not immortalized to minimize the influence of cell culture. Compared to whole organs, they offer the advantage of assaying a pure cell population to avoid the confounding effect of varying cell composition. The composition in CG dinucleotides in the selected species varies between 0.9% (mouse) and 1.4% (rabbit) and all show a genome depletion in CG (CG observed/expected <1) with the mouse being the most depleted species (Fig. 1b). The main DNA methyltransferases (DNMT1, DNMT3A and DNMT3B) are conserved in all selected species while DNMT3L is present only in mammals and absent in chicken (Fig. 1a). RNA-seq quantification indicated that dermal fibroblasts showed a consistent pattern of expression of *Dnmt* genes across species and mainly express *Dnmt1* and *Dnmt3a* (Additional file 1: Fig. S1a).

We generated single-base resolution methylomes by whole genome bisulfite sequencing (WGBS). The average sequencing depth among samples was around 12X after deduplication (Table S1), and 80 to 90% of the CGs in the corresponding reference genome were covered at least 5 times (Additional file 1: Fig. S1b, Table S1). The percentage of unconverted cytosines in non-CG context (CHG and CHH) did not exceed 0.8% for all the selected species (Fig. 1c, lower panel). These values reflect the percentage of unconverted cytosines and the methylation in non-CG context. Therefore, all subsequent analyses were done on CG sites.

### Low genome methylation in chicken compared to mammals

The mean CG methylation level was high in all studied species, varying between 53 and 72% (Fig. 1c, upper panel). Furthermore, the landscape of DNA methylation across genes and flanking regions retained the same shape in all studied species, with a depletion of methylation TSS (Fig. 1d). We noted however that the average methylation in chicken was lower compared to all the mammals (53% in chicken vs 64-72% in mammals, Fig. 1c), and this occurs uniformly in genes and flanking sequences (Fig. 1d). To ensure that this observation is not due to a cell culture bias, we performed WGBS in mouse psoas skeletal muscle and compared with publicly available WGBS data from skeletal muscle samples of different species including chicken (Table S1). The average methylation values in all studied species were slightly higher in muscle compared to primary fibroblasts. Nevertheless, the chicken genome was also less methylated in muscle compared to all mammalian species (61% in chicken vs 70-79% in mammals, Additional file 1: Fig. S1c-d). WGBS methylation data from zebrafish indicate that the zebrafish muscle is hypermethylated at levels equivalent to mammals (Additional file 1: Fig. S1c-d), indicating that reduced genome methylation is not a common characteristic of non-mammalian vertebrates. This agrees with lower global methylation levels observed in chicken forebrain samples compared to mammals and zebrafish [18]. To check whether these patterns are also observed in the germline, we used public WGBS data of sperm from human, mouse, dog, cow and chicken (Table S1) and found that the chicken sperm genome is strongly hypomethylated compared to mammals (Additional file 1: Fig. S1e). Altogether, these results show that the chicken genome shows reduced genome methylation compared to mammals in many cell types.

A particularity of the chicken genome is its low composition in transposable elements compared to the other genomes (13% compared to minimum 40%, Additional file 1: Fig. S1f). We therefore wondered if the lower global methylation in the chicken genome can be attributed to the lower frequency of transposable elements. To test this, we plotted DNA methylation distribution of CGs in different genomic features. While the majority of CGs located in gene promoters were unmethylated in the seven species, the CGs located in the other features (exon, intron, intergenic and transposable elements) were methylated at higher levels in the six mammals compared to the chicken (Fig. 1e). In chicken, the methylation distributions were more relaxed with a high fraction of intermediate methylation levels even for CGs located in transposable elements (Fig. 1e). These observations were confirmed with data from muscle samples (Additional file 1: Fig. S1g). In summary, the chicken genome shows lower methylation levels compared to mammals including at transposable elements, which challenges the view that genome hypermethylation is a hallmark of all vertebrates.

### The mouse has a unique pattern of protection of CpG-rich regions against methylation

**Fig. 2:**
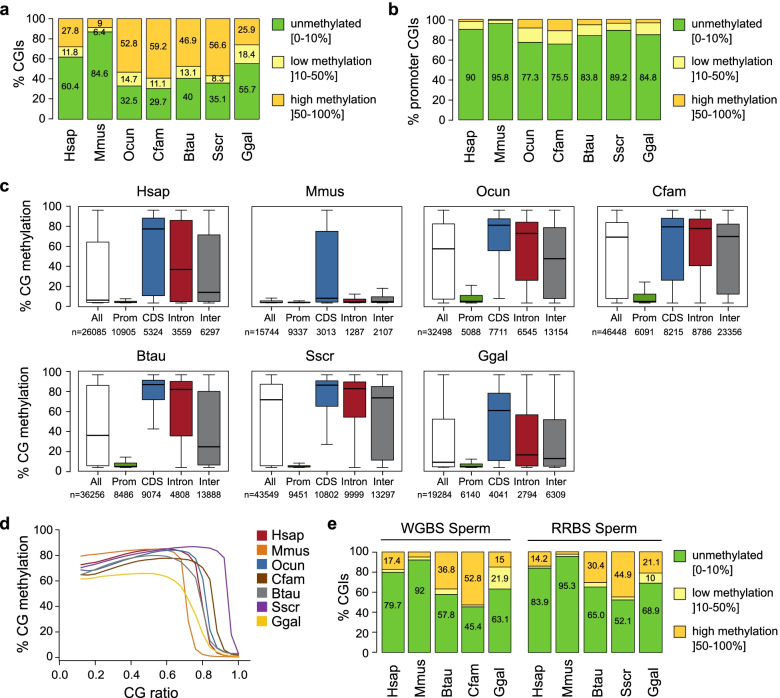
Comparison of DNA methylation of CG-rich regions in vertebrates. **a, b** Stacked bar graphs representing the proportions of CGIs (**a**) and promoter-CGIs (**b**) classified according to their mean CG methylation in dermal fibroblasts. The percent values are indicated on the graphs. **c** Box plots of the mean CG methylation of CGIs overlapping promoters (prom), coding sequences (CDS), introns or intergenic regions (inter) in dermal fibroblasts. The line in the boxplots indicates the median, the box limits indicate the upper and lower quartiles, and the whiskers extend to 1.5 IQR from the quartiles. The numbers of CGIs in each category are indicated below the graphs. **d** Median values of the methylation in 0.5 kb genomic windows according to their CG ratio. **e** Stacked bar graphs representing the proportions of CGIs classified according to their mean CG methylation in sperm samples. Data from WGBS and RRBS libraries are shown

Next, we analyzed DNA methylation of regions with high CpG density. We checked the methylation levels of CGIs regions defined in UCSC tracks with a common threshold of CpG ratio observed/expected above 0.6 for all the studied species. As expected, close to 85% of CGIs in mouse were unmethylated (mean methylation < 10%) compared to 6% low methylated CGIs (mean methylation between 10 and 50%) and 9% highly methylated CGIs (mean methylation > 50%) (Fig. 2a). Surprisingly, we found that the fraction of unmethylated CGIs is much lower in all the other species compared to the mouse, reaching only 30% in rabbit and dog. To check whether this difference among species is related to one specific genomic feature, we annotated the CGIs based on their overlap with TSS, coding sequence (CDS), introns or intergenic regions. This revealed that, while promoter CGIs (pCGIs) are predominantly unmethylated in all species, the percentage of unmethylated pCGIs is higher in the mouse compared to the other species (Fig. 2b). Furthermore, the CGIs in CDS, introns and intergenic regions appear much more methylated in all the other species compared to the mouse (Fig. 2c). These observations were also recapitulated in muscle samples (Additional file 1**:** Fig. S2a-c), demonstrating that they are not restricted to the fibroblasts in culture.

To ensure that this observation is not biased by CGI annotations and further explore the relationship between CpG density and methylation, we correlated DNA methylation and CpG ratio observed/expected in 0.5 kb genomic windows for each species. In the mouse, most of the windows with a CpG ratio above 0.7 are hypomethylated. In contrast, we found that the probability of methylation decreases at a higher CpG ratio in all the other species (Fig. 2d). This is consistent with a previous study showing that experimentally defined hypomethylated islands have a much lower CpG ratio in the mouse compared to the human genome [19]. Altogether this shows that the limit of CpG ratio that protects against methylation varies between species and is lower in the mouse compared to other vertebrates.

Having identified a higher fraction of methylated CGIs in somatic cells in all the studied species compared to the mouse, we wondered whether CGIs also show an increased frequency of methylation in the germline. We used public WGBS and Reduced Representation Bisulfite Sequencing (RRBS) data of sperm from human, mouse, dog, cow and chicken and complemented this set by performing RRBS on sperm from human, pig and chicken (Table S1 and S2). As in somatic tissues, this revealed that the fraction of methylated CGIs in sperm is higher in other species compared to the mouse (Fig. 2e, Additional file 1: Fig. S3a-b). Additionally, we analyzed public WGBS profiles in oocytes of human, mouse and cow and found again an increased frequency of methylation of annotated CGIs in human and cow compared to the mouse (Additional file 1: Fig. S3c). In each species, the CGI methylation status in fibroblasts positively correlates with CGI methylation in gametes (Additional file 1: Fig. S3d), suggesting a consistent pattern of CGI methylation between gametes and somatic cells. In summary, this shows that CG-rich sequences are more frequently methylated in germ and somatic cells of other vertebrates compared to the mouse.

### Large unmethylated valleys are conserved among vertebrates

Large unmethylated regions covering several kilobases have been previously described in human and mouse and named DNA Methylation Valleys (DMVs) or canyons [10–12]. These DMVs often cover entire gene bodies and are enriched in genes encoding transcription factors and developmental regulators [10, 11]. We therefore examined whether DMVs are a conserved feature of vertebrate methylomes.

**Fig. 3:**
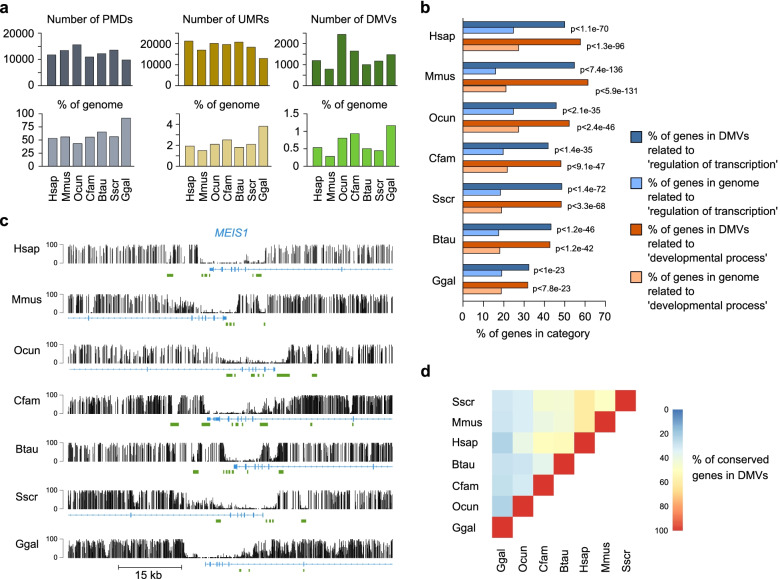
Conservation of DNA Methylation Valleys (DMVs) across vertebrates. **a** Bar graphs showing the number of identified PMDs, UMRs and DMVs (UMR >= 5kb) and the percentage of the genome covered by each feature in dermal fibroblasts of the seven studied species. **b** Gene ontology analysis of genes located in DMVs. The graph shows the enrichments of ontology terms 'regulation of transcription' and 'developmental process' in DMV-associated genes compared to all genes (*p* value: hypergeometric test). **c** Genome browser snapshots of WGBS methylation scores showing a conserved DMV overlapping the *MEIS1* gene in dermal fibroblasts of the seven species. Each WGBS track shows the percent methylation of individual CpGs between 0 and 100%. CpG islands (green rectangles) and Ensembl gene annotations are shown below the tracks. **d** Analysis of the overlap of genes located in DMVs in fibroblasts across the seven vertebrate species. Each square in the heatmap represents the percentage of common genes associated with DMVs between two species. Details about the calculation are provided in the Methods section

To identify DMVs from the dermal fibroblast WGBS datasets, we used MethylSeekR [20]. MethylSeekR first identified partially methylated domains (PMDs), which were found to cover a large portion of the genome (50-75%) in all the mammals (Fig. 3a). Notably, the percentage of the genome covered by PMDs was higher in chicken fibroblasts (90%), consistently with the reduced global methylation of the chicken genome (Fig. 3a). After excluding the genomic regions that contain PMDs, MethylSeekR identified similar numbers of unmethylated regions (UMRs) in each species (Fig. 3a). We subsequently defined DMVs as UMRs with a size > 5kb, which led to the identification of 700 to 2400 DMVs in primary fibroblasts from the seven vertebrates, occupying 0.3 to 1.2% of the genome (Fig. 3a). DMVs ranged in size from 5 kb to ~60 kb. Gene ontology analysis showed that DMVs are strongly enriched for genes related to developmental processes and regulation of transcription in all studied species (Fig. 3b), such as *HOX*, *DLX*, *LHX*, *FOX*, *GATA*, *ZIC*, *KLF* and *TBX* transcription factors (Table S3). Figure 3c shows an example of conserved DMV overlapping the *MEIS1* gene in all species. To evaluate this conservation, we performed a pairwise analysis of the common genes associated with DMVs between each species and found a high propensity for orthologous genes to have DMVs (Fig. 3d). These results show that large unmethylated valleys covering transcription factor genes and developmental genes are highly conserved among vertebrates.

### Prediction of allele-specific methylation reveals a conserved set of imprinted genes in mammals

**Fig. 4:**
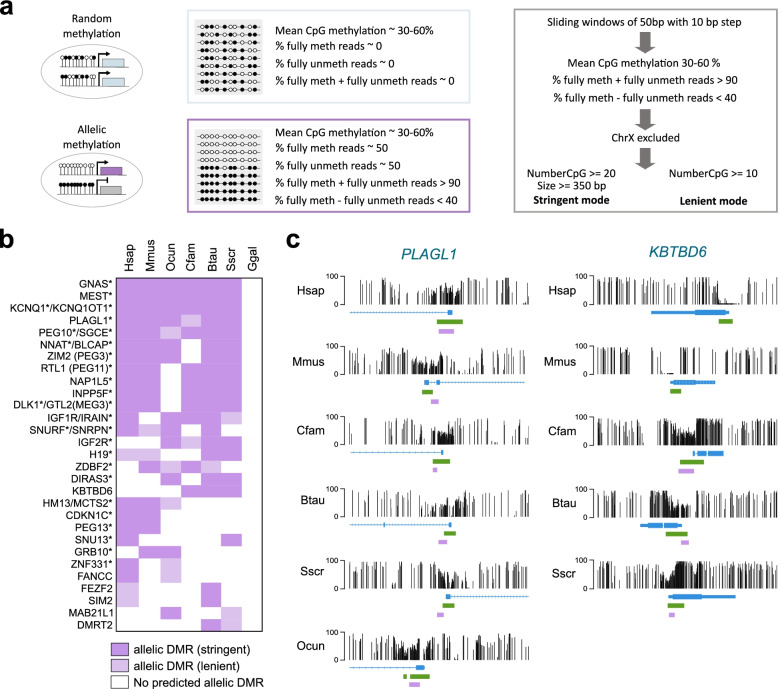
Prediction of imprinted DMRs in mammals using WGBS data. **a** Description of the pipeline used to detect potential imprinted DMRs using WGBS data. To differentiate allelic from partial random methylation, we use single read methylation scores to identify regions that contain a mixture of fully methylated and fully unmethylated reads. We applied a stringent mode to identify regions larger than 350 bp with a minimum of 20 CpGs, and a lenient mode to identify regions with a minimum of 10 CpGs. **b** Top ranked genes associated with predicted allelic DMRs in fibroblast in stringent and lenient mode in at least 2 species. Asterisks indicate genes previously known to be imprinted in human or mouse. **c** Genome browser snapshots of WGBS profiles over the *PLAGL1* (left panel) and *KBTBD6* (right panel) genes in dermal fibroblasts of 6 mammalian species. *KBTBD6* is not shown in the rabbit because of lack of gene annotation. CpG islands (green rectangles), predicted DMRs (purple rectangles) and Ensembl or Refseq gene annotations are shown below the tracks

Imprinted genes are under control of germline differentially methylated regions (gDMRs), which acquire differential methylation in the parental gametes and can also direct the establishment of somatic DMRs in the embryo. Imprinted DMRs are CpG-rich and present ~50% methylation because either the maternal or paternal allele is highly methylated and the other one is unmethylated. Furthermore, they are generally maintained in all somatic tissues and thus can be used to comprehensively identify imprinted genes irrespective of whether they are expressed. The catalog of imprinted DMRs is well described in mouse and human, but to what extent imprinted genes are conserved in all mammals remains elusive. We therefore wished to use the WGBS data to predict imprinted DMRs and investigate their conservation across mammals. We developed a pipeline to predict regions of allelic methylation that have a mean methylation between 30 and 60%, more than 90% of either fully methylated and unmethylated reads and a maximum of 40% difference between fully methylated and unmethylated reads (Fig. 4a, see Methods). We excluded the regions on the X chromosome as they can be subjected to X chromosome inactivation in females, and those overlapping developmental genes (such as *HOX* and *TBX*) previously known to have variable allele specific methylation [21, 22]. Finally, we added stringent criteria by selecting only regions with more than 20 CpG and bigger than 350 bp (stringent mode) while keeping a lenient prediction mode with only a selection for regions with more than 10 CpG (Fig. 4a).

When we applied this pipeline prediction to mouse fibroblasts, 18 out of the 20 known mouse gDMRs were identified and 30 out of the 33 identified regions under stringent criteria were close to a known imprinted gene (Table S4), which demonstrates the reliability of the pipeline. Applying this pipeline to the six mammals led to the identification of 29 genes close to regions with allelic methylation in at least 2 species (Fig. 4b). The top 16 ranked genes predicted in at least 4 species were known imprinted genes in the mouse, such as *MEST, GNAS, PEG10*, *KCNQ1* and *PLAGL1* that were predicted to be imprinted in all tested mammals (Fig. 4b). None of these DMRs were identified in the chicken known to lack genomic imprinting (Fig. 4b). Thus, this analysis reveals a conserved core set of genes predicted to carry imprinted methylation in mammals.

Conversely, we also make predictions of novel DMRs occurring in mammals other than mouse and human. One example is *KBTBD6,* a gene not previously described as imprinted in mouse or human (Fig. 4b, c). In our pipeline, this gene is predicted for allelic methylation in dog, cow and pig. Interestingly, it has been recently identified as an imprinted gene in pig with an allelic expression screening strategy [23].

In the mouse, ZFP57 interacts with a CpG-methylated hexanucleotide (TGCCGC) in gDMRs and is required for the maintenance of allele-specific methylation during development [24, 25]. To investigate whether conserved mechanisms take place in mammals, we performed an enrichment analysis of transcription factor (TF) motifs from the JASPAR database in the predicted stringent allelic DMRs of each species. We selected motifs present in more than 50% of DMRs with a *p*-value < 0.01 compared to random regions with similar GC content. The ZFP57 motif showed a significant enrichment in DMRs of all mammalian species except the dog, suggesting a conserved role in maintaining imprinted DMRs across mammals (Table S5). Interestingly, another zinc finger protein ZBTB14 showed a motif enrichment in five mammalian species, suggesting a potential role in regulating imprinted DMRs (Table S5). These data suggest potential conservation of the molecular mechanisms regulating imprinted allelic methylation across mammals.

### Reconfiguration of DNA methylation is a hallmark of X-chromosome inactivation in all mammals

**Fig. 5:**
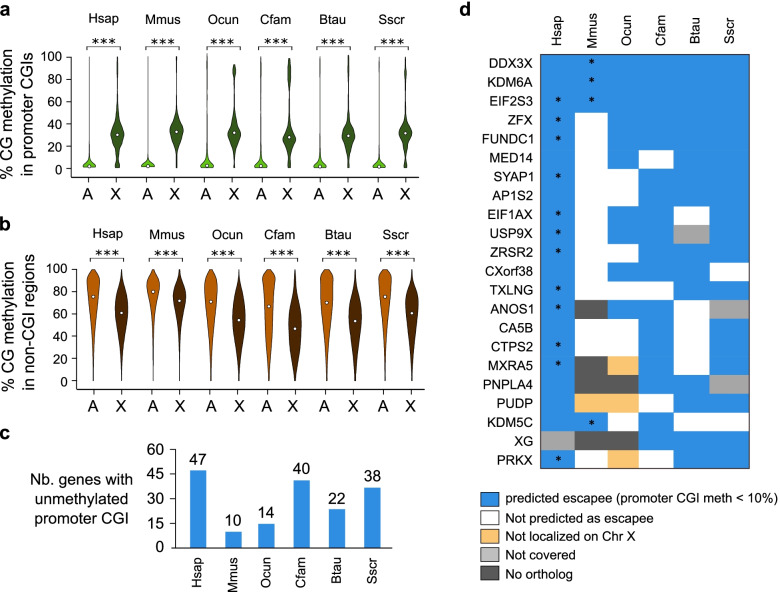
Conserved DNA methylation signature of X chromosome inactivation in mammals. **a** Violin plots of CG methylation scores of promoter-CGIs measured by WGBS in dermal fibroblasts across autosomes (A) or the X chromosome (X) in mammalian species. Median values are indicated by white circles. ***: *p*-value < 0.001 (Wilcoxon test). **b** Violin plots of CG methylation scores of 1 kb genomic tiles (excluding CGIs) measured by WGBS in dermal fibroblasts across autosomes (A) or the X chromosome (X) in mammalian species. Median values are indicated by white circles. ***: *p*-value < 0.001 (Wilcoxon test). **c** Number of X-linked genes with an unmethylated promoter CGI (methylation < 10%), predicted to escape XCI, identified in each species. **d** Table of genes predicted to escape XCI in at least 3 species. Asterisks indicate genes previously shown as escapees in human and mouse

DNA methylation is reconfigured on the inactive X chromosome in human and mouse. Promoter CGIs are usually unmethylated on the active X chromosome (Xa) and highly methylated on the inactive X chromosome, leading to an average methylation level of 30-40% [26, 27]. In humans, an early study also showed that the active X is more methylated than the inactive X chromosome in gene bodies [28]. We took advantage of using female dermal fibroblasts in all species to study the conservation of DNA methylation changes associated with X-chromosome inactivation across placental mammals. For each species, we compared the mean CG methylation of promoter-CGIs and non-CGI regions (1 kb tiles) on the X chromosome and autosomes. As expected, in human and mouse, promoter-CGIs on autosomes were unmethylated while the major fraction of promoter-CGIs on the X chromosome had a mean methylation around 30% (Fig. 5a). This pattern was recapitulated in all the analyzed mammalian species (Fig. 5a). Conversely, the mean methylation of non-CGI regions was significantly lower on the X chromosome compared to autosomes in all mammals (Fig. 5b). Interestingly, the global hypomethylation of non-CGI regions is more drastic in all other mammals compared to the mouse (Fig. 5b). This indicates that the DNA methylation signature of X chromosome inactivation is conserved in mammals.

Promoter CGI methylation is strongly predictive of the XCI status and unmethylated pCGIs can be used to predict genes that escape XCI [26, 29]. For each species, we determined X-linked genes with unmethylated pCGI (<10%) that presumably escape XCI in order to investigate the conservation of XCI escape calls across species. We refined this analysis by manually checking on the genome browser potential promoter CGIs that could not be identified due to incorrect gene annotation. Mouse showed the lowest number of XCI escapees (Fig. 5c), which is in agreement with a recent study [30]. It is important to note that the number of genes escaping XCI in rabbit is underestimated due to poor gene annotation in this species. Indeed, we identified in rabbit several unmethylated CGIs that colocalized with a transcription start but without gene annotation. Overall, we identified 22 genes escaping XCI in at least three mammalian species (Fig. 5d). *DDX3X*, *KDM6A, EIF2S3* were predicted XCI escapees in all the studied mammals, while most other genes were predicted as XCI escapee in mammals other than the mouse.

Altogether, these results reveal conservations of DNA methylation patterns associated with XCI in mammals with the mouse being an outlier in terms of hypomethylation of non-CGI regions and the number of XCI escapees.

### Correlation between DNA methylation and gene expression

Next, we focused on the relationship between DNA methylation and gene transcription. Gene bodies represent the most conserved targets of DNA methylation in eukaryotes [1, 13] and in mouse and human, high gene body methylation has been associated with expressed genes [31–33]. To test whether this applies to other species, we quantified gene expression in the primary fibroblasts by RNA-seq (Table S6). In all mammals, genes with high expression (log2 rpkm > 0) were more likely to have high gene body methylation (Additional file 1: Fig. S4a-b). Compared to the other mammals, the mouse was again an exception with a lower difference in gene body methylation between highly expressed and lowly expressed genes. Surprisingly, we did not observe the same tendency in the chicken (Additional file 1: Fig. S4a-b).

**Fig. 6:**
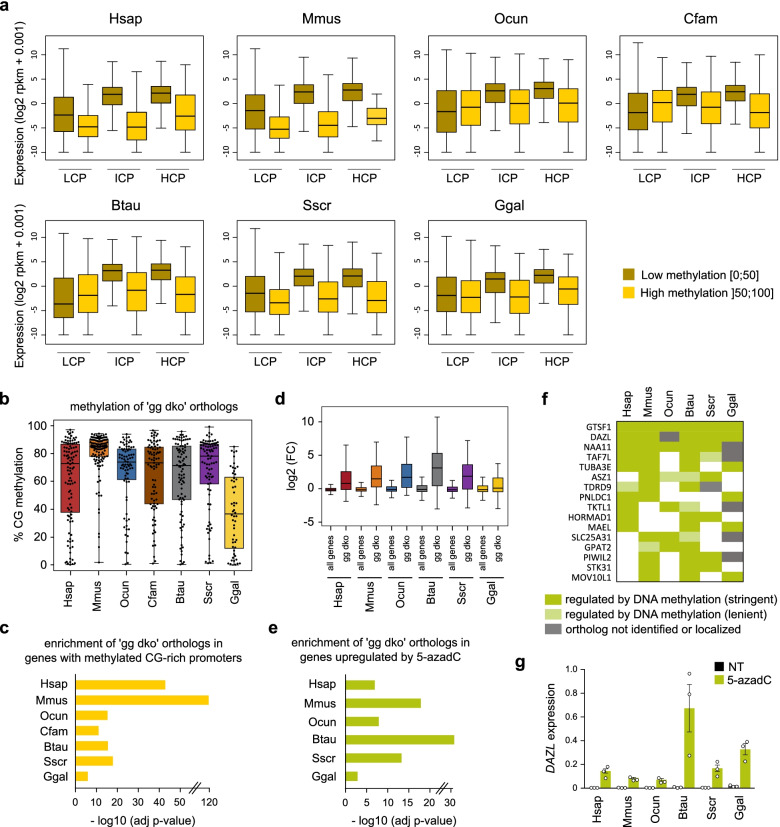
Impact of promoter DNA methylation on gene expression in vertebrates. **a** Boxplots showing gene expression scores (rpkm) depending on the level of promoter DNA methylation for genes with LCP, ICP or HCP promoters in each species. **b** Boxplots of promoter DNA methylation scores in fibroblasts for the previously identified list of germline genes upregulated in *Dnmt3a/3b* double knockout embryos (termed 'gg dko' genes). For the species other than mouse, orthologs of mouse 'gg dko' genes are shown. **c** Enrichment of 'gg dko' orthologs among genes with methylated CG-rich promoters in fibroblasts for each species. The graph shows the associated adjusted *p*-values (-log10) calculated by hypergeometric tests. **d** Boxplots of the fold change (FC) of gene expression of 'gg dko' orthologs compared to all genes after 5-azadC treatment in fibroblasts. **e** Enrichment of 'gg dko' orthologs among genes upregulated by 5-azadC in each species. The graph shows adjusted *p*-values (-log10) calculated by hypergeometric tests. **f** Table showing germline genes upregulated by 5-azadC in at least 3 vertebrate species. The stringent mode corresponds to genes with a methylated promoter in control condition (> 50%), a fold change upon 5-azadC treatment > 3 and an adjusted *p*-value < 0.01. The lenient mode corresponds to less stringent cut-offs on promoter DNA methylation (> 25%) or fold change upon 5-azadC treatment (> 2). Genes in white did not pass the previous criteria. **g** RT-qPCR quantification of the expression of the *DAZL* gene in dermal fibroblasts treated with 5-azadC for 72h compared to untreated fibroblasts (NT). The expression was normalized to two housekeeping genes (*Gusb* and *Mrpl32*) (mean ± SEM, *n*=3 independent experiments). In the boxplots, the line indicates the median, the box limits indicate the upper and lower quartiles, and the whiskers extend to 1.5 IQR from the quartiles in a, d or to the data extremes in b

To investigate the relationship between gene expression and promoter DNA methylation, we classified gene promoters into three groups based on their CG ratio: low (LCP), intermediate (ICP) and high (HCP) CG ratio promoters with an adjustment of CG ratio for each species (Additional file 1: Fig. S5a). In all species, HCP promoters were mostly hypomethylated, whereas LCP promoters were in majority highly methylated and ICP promoters showed intermediate levels of methylation (Additional file 1: Fig. S5b). In line with our above CGI methylation data, we noted that the mouse had the lowest proportion of highly methylated HCPs and ICPs (Additional file 1: Fig. S5b). Comparing RNA-seq expression and promoter DNA methylation revealed a significant anti-correlation between gene expression and promoter methylation for ICPs and HCPs in all species (Fig. 6a). This anticorrelation was less marked in some species such as rabbit and dog, probably due to more frequent inaccurate gene annotations in these species. In contrast, LCPs showed an anticorrelation in human, mouse and pig but not in the other species. Altogether these results demonstrate that methylation of CpG-rich promoters correlates with low gene expression across vertebrates.

### Repression of germline genes and ERVs are conserved functions of DNA methylation across vertebrates

Having shown that methylation of CpG-rich promoters correlates with gene silencing, we investigated which genes are principal targets of DNA methylation-mediated repression. In the mouse, repression by DNA methylation of CpG-rich promoters occurs predominantly at germline genes, but it is unknown if this function is conserved in other vertebrates. Interestingly, GO enrichment analysis showed that most of the top ranked biological process terms associated with highly methylated (methylation > 50%) CpG-rich promoters (ICPs and HCPs) relate to germline functions (reproduction, meiosis, piRNA process, gamete generation...) in all mammals (Additional file 1: Fig. S5c, Table S7). In chicken, although the top ranked terms were not related to germline functions, many germline gene orthologs (such as *DAZL, MEIOC, MAEL, DMRTB1, PNLDC1, RBM46* … ) were highly methylated but listed in different enriched terms (Table S7). Compared to the mouse, germline GO terms were less enriched in the other species, which is consistent with our above results showing more frequent methylation of CpG-rich promoters in other species. To avoid biases due to incomplete gene ontology annotation, we focused on a subset of germline genes that we previously identified as the first targets of DNA methylation in mouse double knockout (dko) embryos lacking DNMT3A and DNMT3B (hereafter termed 'gg dko' for 'germline genes dko') [6]. In all the studied species, we found that the orthologs of these genes (Table S8) tend to have methylated promoters (Fig. 6b) and are significantly enriched among genes with highly methylated CpG-rich promoter (Fig. 6c). These data demonstrate that these germline gene promoters are conserved targets of DNA methylation in vertebrates.

To test for a causal link between promoter DNA methylation and repression of germline genes, we treated proliferating primary dermal fibroblasts of each species with the DNA methylation inhibitor 5-azadeoxycytidine (5-azadC) for 72 hours. To validate the effect of 5-azadC, we performed RRBS (Table S2) and confirmed reduced global DNA methylation in human, mouse, rabbit, cow, pig and chicken 5-azadC-treated cells (Additional file 1: Fig. S6a). In contrast, we could not achieve DNA methylation inhibition by 5-azadC treatment in the dog fibroblasts because they immediately stopped dividing upon treatment. Transcriptomic analysis by RNA-seq revealed that 5-azadC treatment led to more upregulated than downregulated genes in the six species (absolute fold change > 3 and adjusted *p*-value < 0.01, Table S9), which is consistent with a DNA methylation inhibition effect (Additional file 1: Fig. S6b). Interestingly, we found that ‘gg dko’ orthologs have high fold changes of expression compared to the whole gene population in all species (Fig. 6d), and were significantly enriched among genes upregulated by 5-azadC treatment in the 6 studied species (Fig. 6e). Furthermore, 'gg dko' orthologs were also enriched among upregulated genes when we used only genes with a methylated CpG-rich promoter as background in the 6 studied species (Fig. S6c), indicating that germline genes are preferentially affected by 5-azadC among all the methylated genes. We checked whether a common set of germline genes is repressed by DNA methylation and found 16 germline genes upregulated by 5-azadC treatment in at least 3 species (Fig. 6f). Among these genes, *DAZL* was found upregulated in 5 tested species (Additional file 1: Fig. S6d), which was validated by RT-qPCR (Fig. 6g). Although *DAZL* is not annotated in rabbit, we designed primers for a region supposed to be the ortholog of *DAZL* 3’UTR and observed an induction of this potential transcript upon 5-azadC treatment (Fig. 6g). These results show that germline genes are conserved targets of DNA methylation-mediated repression in vertebrates.

Finally, we investigated whether DNA methylation has a conserved role in repressing transposable elements. To this end, we counted unique and multiple-mapping reads in RepeatMasker annotations to evaluate the expression of TE families upon 5-azadC treatment in each species. As expected, we found a high number of upregulated TEs in mouse fibroblasts, including numerous Intracisternal A particles (IAP) families and other *LTR-containing endogenous retroviruses (ERVs)* (Additional file 1: Fig. S7). Several TE families were also found significantly upregulated upon 5-azadC treatment in all other species, which belong mostly to *LTR-containing* ERV classes (Additional file 1: Fig. S7). The number of upregulated TE families was higher in mouse compared to the other species. This might be attributed to the presence of more evolutionary young ERVs (such as IAPs) in the mouse genome, or to differences in the response to 5-azadC treatment or quality of genome annotations. Interestingly, this analysis revealed a high number of upregulated ERV families in chicken, indicating that although DNA methylation is globally reduced in the chicken genome, it is nevertheless involved in maintenance of ERV repression.

Altogether, these results show that repression of germline genes and ERVs are evolutionary conserved functions of DNA methylation among vertebrates.

## Discussion

In this study, we evaluated the conservation of the distribution and functions of DNA methylation among vertebrates. While our current knowledge about DNA methylation stems mainly from the mouse, it is important to assess to which extent it is a representative model for the study of DNA methylation. We therefore selected species that represent important biomedical and agronomic models (rabbit, dog, cow, chicken, pig). Importantly we investigated DNA methylation in a comparable and homogenous cell population of the same sex in all species.

First, we noted an expected conservation of global DNA methylation distribution in vertebrates. As previously shown, conserved properties include high global CG methylation (ranging from 60 to 80%) except around gene TSS, and higher methylation of intragenic sequences compared to intergenic sequences. Despite widespread DNA methylation, large constitutively hypomethylated regions preferentially marked by H3K27me3 and known as DNA methylation valleys (DMVs) have been described in mouse, human and zebrafish [10–12]. Extending these previous studies, we show that DMVs are conserved across vertebrates and enriched for developmental genes in all tested vertebrates. The exact functions of DMVs at developmental genes are unknown. It has been suggested that hypomethylation may reduce the risks of deamination mutations caused by DNA methylation especially that coding regions of developmental transcription factors including *HOX* genes are enriched in CpGs [34]. Alternatively, hypomethylation may be crucial to protect transcription factor binding sites and ensure the plasticity of gene expression in development. The mechanisms underlying the protection of DMVs against methylation are not fully understood. In mouse, it has been shown that Polycomb regulates DNA methylation in DMVs by recruiting TET proteins [12] and a recent report demonstrated the role of an uncharacterized gene *QSER1* along with *TET1* in protecting DMVs in human embryonic stem cells [35]. Investigating the conservation of these mechanisms among vertebrates would be of great interest in the future.

Our findings also highlight interesting discrepancies among vertebrate methylomes. We show that the chicken genome has reduced global methylation in somatic cells and even more drastically in sperm compared to mammals. This is consistent with brain methylation data from a recent study [18] showing reduced genome-wide methylation levels in two bird species (chicken and great tit) compared to mammals. In the future it will be interesting to investigate DNA methylation in other bird species to determine whether reduced methylation is a characteristic of bird genomes. The causes of this reduced methylation are currently unknown. DNMT3L is absent in chicken as DNMT3L was gained by gene duplication in the common amniote ancestor and then lost during the evolution of the bird and monotreme lineage, which could contribute to low sperm DNA methylation [36]. However, the hypermethylation observed in platypus brain (a monotreme species) argues against an impact of the absence of DNMT3L on the methylome of somatic tissues [18]. Interestingly, birds maintain a small genome size to ensure a high cell metabolism [37]. It has been proposed that this occurs by extensive DNA loss to counteract the expansion of TE sequences [38]. It could be hypothesized that the reduced global methylation of bird genomes facilitates DNA recombination while still participating in the maintenance of TE repression. Another divergence of the chicken genome compared to mammals is observed within gene bodies considered to be the most common target for methylation among eukaryotes [1, 13]. Strikingly, in contrast to mammals, expressed genes in chicken were not associated with higher gene body methylation. This raises the question whether the affinity of the PWWP domains of the *de novo* DNA methyltransferases (DNMT3A and DNMT3B) with H3K36me3 is different in chicken compared to mammals.

We uncovered important discrepancies between the mouse and other mammalian species. First, we observed a much stronger protection of CpG-rich regions against methylation in the mouse genome compared to all other studied species. This is in agreement with a recent study reporting a higher frequency of methylation of CpG-rich regions in human cells compared to mouse cells [39]. Furthermore, previous studies also reported a greater trend toward birth of new hypomethylated regions in rodent sperm [14] and a higher frequency of hypomethylated domains in mouse oocytes compared to human, pig and cow [40]. Interestingly, introduction of the human chromosome 21 in the mouse leads to the appearance of many novel hypomethylated regions with high CpG density on the human chromosome 21 [41]. This suggests that the increased protection of CpG-rich sequences in the mouse is not encoded in the DNA sequence but depends on intrinsic protection pathways in the mouse.

The mouse was also an outlier in terms of the number of genes predicted to escape XCI. X escapees have different signatures compared to genes subjected to XCI, including biallelic expression, enrichment of active and depletion of repressive histone marks, hypermethylation in gene bodies and reduced levels of promoter DNA methylation [42]. Here we limited our search for XCI escapees to genes with unmethylated promoter CGI. Although the escape from XCI can vary between tissues or age [43], the fact that the mouse is an outlier is an agreement with a recent study [30]. Our results also match gene expression studies that predicted few escapees (3-7%) in mouse tissues [43] compared to 23% in human tissues [44]. Given the importance of XCI escape for human sexually dimorphic disease risk [45], this highlights the importance of finding relevant species to model XCI escape. Similarities between human and mammalian non-rodent animals can direct toward more appropriate biomedical models.

In this study we used WGBS data to investigate the conservation of genes subjected to parental genomic imprinting in mammals. Different approaches have been used to predict imprinted genes genome-wide: genome-wide screens for parental-specific methylation using tissues from biparental origins [46, 47], computational analyses of specific DNA sequence features [48], or analysis of allelic expression bias from RNA-seq experiments on reciprocal crosses [49]. However, these approaches have been mostly limited to mice because of the necessity of complex breeding schemes. Therefore, few studies investigated the conservation of imprinting in other mammals, mostly resulting from candidate gene analysis of homologs of known mouse imprinted genes. Using our pipeline to identify allele specific methylation, we performed the first comparative analysis of imprinted DMRs in six mammals. Although transcriptomic data would be essential to verify the parental imprinting, our approach allows a reliable prediction of imprinted DMRs as demonstrated by the fact that 30 out of 33 predicted DMRs in the mouse are linked to known imprinted genes. Our data highlight that many predicted DMRs are common to most of the studied mammals, in particular a core set of genes (including *MEST, GNAS, PEG10, SGCE, PLAGL1, NAP1L5, PEG3, KCNQ1, NNAT, INPP5F, RTL1, IGF2R, SNRPN, H19*) with a predicted nearby DMR in at least 4 out of 6 mammals. Lack of prediction of DMRs in some species may be due to technical problems such as biased PCR amplification or lack of WGBS coverage, as exemplified by the mouse gDMR of *Igf2r and Rasgrf1* that were not predicted in our analysis due to lack of coverage. Our data support the model that imprinting of a core set of genes was established in early eutherian ancestors while imprinting of other genes has arisen subsequently. This coincides with the timing of emergence of novel CGIs at many DMRs in the eutherian ancestor [50]. Using motif analysis in predicted DMRs, we suggest a conserved role of ZFP57 in genomic imprinting and also identify the zinc finger protein ZBTB14 as a potential regulator of imprinting. Published RNA-seq datasets indicate that *Zbtb14* expression is detectable in oocytes or early embryos in mouse, human, pig and cow, which is compatible with a possible function of *Zbtb14* in genomic imprinting during early development. Functional experiments will be needed to investigate whether *Zbtb14* has a role in genomic imprinting.

Last, we show that the repression of germline specific genes by DNA methylation is conserved among the studied species. Germline genes are the preferential targets of DNA methylation of CpG-rich promoters in the mouse [8, 51]. Although DNA methylation of CpG-rich promoters is less infrequent in the other species, we found that germline genes remain the preferential targets in all tested vertebrates. Our conclusions may also apply to zebrafish as suggested by a recent study in which the authors show the existence of a common set of germline specific genes that become methylated during zebrafish and mammalian embryogenesis [52]. Furthermore, germline genes are enriched among methylated genes upregulated by 5-azadC in all tested species. Thus, our study demonstrates that repression of germline genes is a prime conserved function of DNA methylation and an ancient regulatory mechanism in vertebrates. This agrees with sparse evidence from the literature suggesting that DNA methylation suppresses expression of the germline gene *Dazl* in pig and chicken [53–55]. Why DNA methylation has evolved as the prime mechanism to suppress the expression of a set of germline genes in vertebrates is unclear. One possibility is that this evolved as a mechanism to couple epigenetic reprogramming with robust expression of meiotic and piRNA defense genes in the germline.

## Conclusions

Our study provides a detailed analysis of the conservation of DNA methylation patterns and functions across vertebrates. We reveal conserved functions of DNA methylation in gene and transposon regulation, which highlights the roles of this epigenetic mark in vertebrates. We also reveal differences between the mouse and other vertebrates, indicating that caution should be taken when extrapolating results from DNA methylation studies in the mouse to all vertebrates.

## Methods

### Biological samples

Mouse dermal fibroblasts were derived from skin of a 4 days old C57BL/6J mouse. Human dermal fibroblasts were purchased from CellBiologics (#H6068, fibroblasts from skin of a 40-year-old female donor). Rabbit dermal fibroblasts were a gift from N. Daniel and V. Duranthon and were derived from an ear skin biopsy of an adult female New Zealand white rabbit. Dog skin fibroblasts were purchased from Coriell (#AG08056, fibroblasts from abdomen skin of a 4-year-old female). Bovine skin fibroblasts were purchased from Coriell (#GM03655, fibroblasts from skin of a 5-year-old Holstein female). Pig skin fibroblasts were derived from a skin biopsy of an adult female Large White pig. Chicken dermal fibroblasts were derived from a skin biopsy of an adult female chicken. All fibroblasts were tested negative for mycoplasma. Sperm samples were collected from an adult Large White pig and an adult chicken rooster. The human semen sample was obtained from a volunteer 35-year-old man with normal semen parameters at spermiogram (count, progressive motility and vitality) according to World Health Organization’s criteria (5th Edition of the WHO Laboratory Manual for the Examination and Processing of Human Semen (2010)). This sample was selected as no leucocytes nor others cells were identified in order to limit diploid cell contamination.

### Cell culture and 5-azadC treatment

All fibroblasts were grown in DMEM-GlutaMax supplemented with 10% fetal bovine serum and 50U/mL penicillin-streptomycin in a humidified atmosphere containing 5% CO2 at 37°C. 5-azadC was purchased from Sigma (A3656) and prepared in water at 1mg/mL stock concentration. Cells were treated with 1 μM final concentration of 5-azadC for 72 hours with medium renewal every day. The treatment was performed 3 times independently for each species.

### Nucleic acid extraction

DNA and RNA were extracted using the Allprep DNA/RNA Kit (Qiagen) and quantified using the Qubit 2.0 fluorometer (ThermoFisher scientific). Integrity was checked by gel electrophoresis.

### Preparation of WGBS libraries

WGBS libraries were prepared as described previously [56]. Briefly, 100 ng of genomic DNA were fragmented to 350 bp using a Covaris E220 sonicator. Bisulfite conversion was performed with the EZ DNA Methylation-Gold kit (Zymo), and WGBS libraries were prepared using the Accel-NGS Methyl-Seq DNA library Kit (Swift Biosciences) following the manufacturer’s instructions. 4 to 6 cycles were used for the final PCR amplification of the WGBS libraries. The libraries were purified using Agencourt AmpureXP beads (Beckman-Coulter) and sequenced on an Illumina HiSeq 4000 sequencer by Integragen SA (Evry, France) to produce 100 bp paired-end reads.

### Preparation of RRBS libraries

RRBS libraries were prepared as described [56]. Briefly, 100 ng genomic DNA were digested by MspI (Thermo Scientific), end-repaired and A-tailed with Klenow fragment exo- (Thermo Scientific), then ligated to methylated adapters with T4 DNA ligase (Thermo Scientific). Size selection was performed by gel excision to select fragments ranging from 150 to 400 bp. DNA was purified using the MinElute gel extraction kit (Qiagen) and bisulfite-converted twice with the EpiTect bisulfite kit (Qiagen) following the manufacturer’s instructions. The final libraries were amplified using the Pfu Turbo Cx hotstart DNA polymerase (Agilent) with 12 to 14 PCR cycles, purified using Agencourt AmpureXP beads (Beckman-Coulter) and sequenced on an Illumina HiSeq 4000 sequencer by Integragen SA (Evry, France) to produce 75 bp paired-end reads.

### Processing of WGBS sequencing reads

Low quality bases as well as the first five bases of reads R1 and ten bases of reads R2 and adapter sequences were trimmed with Trim Galore v0.4.4 (parameters -q 20 --clip_R1 5 --clip_R2 10 --stringency 2). Reads were mapped to the corresponding genome and cleaned for duplicates using Bismark v0.22.1 with default parameters. Reads with incomplete bisulfite conversion were removed using the filter_non_conversion tool in Bismark with the parameters --minimum_count 5 and --percentage_cutoff 50. Methylation calls were extracted as the ratio of the number of Cs over the total number of Cs and Ts using the Bismark_methylation_extractor. Only CpGs with a minimum sequencing depth of 5X were included in the analyses. Public WGBS sequencing data were remapped using the same pipeline, except for the human and cow oocyte datasets for which we used the methylation calls provided by the authors.

### Processing of RRBS sequencing reads

Reads were trimmed with Trim Galore (v0.4.4) to remove adapter sequences and low-quality ends with a Phred score below 20. Trim Galore was run in –non_directional and –rrbs mode to remove two additional bases artificially introduced at the MspI restriction sites. Sequencing reads were mapped to the corresponding genome with Bismark v0.18.2 with default parameters. A maximum of two mismatches and an insertion size for paired-end sequences of between 30 and 400 bp were allowed. Methylation scores were extracted as the ratio of the number of Cs over the total number of Cs and Ts using the Bismark_methylation_extractor. CpG methylation ratios from both strands were combined and filtered for a minimum sequencing depth of 10X. The bisulfite conversion efficiency was estimated by calculating the C to T conversion at the end-repaired MspI CpG sites, which was in most cases greater than 99% (Table S2).

### Genome annotations and data analysis

We used the genome assemblies human hg38, mouse mm10, rabbit oryCun2, dog canFam3, cow bosTau8, pig susScr11, chicken galGal6, and Ensembl gene annotations Mus_musculus.GRCm38.87, Homo_sapiens.GRCh38.87, Oryctolagus_cuniculus.OryCun2.0.99, Canis_familiaris.CanFam3.1.89, Bos_taurus.UMD3.1.94, Sus_scrofa.Sscrofa11.1.94 and Gallus_gallus.GRCg6a.95. CpG islands annotations were obtained from the UCSC Genome Browser. CGIs were annotated as promoter CGIs if they overlap a TSS, CDS CGIs if they overlap a CDS but not a TSS, intron CGIs if they overlap an intron but not a CDS nor TSS, and intergenic CGIs if they do not overlap a TSS, CDS nor intron. Due to the low coverage of oocyte WGBS datasets, CGIs were filtered to have a minimum of 35% covered CpGs for DNA methylation analysis in oocytes. Promoters were defined as regions ranging from -1000 to +500 bp from annotated TSS. The promoter CpG ratio was calculated in the 1500 bp window using the following formula: (number of CpGs x number of bp) / (number of Cs x number of Gs). In case of multiple promoters for one gene, we selected the one with the highest CpG ratio. For the promoter classification, we plotted the distribution of CG ratios for all promoters and chose cut-offs to define LCP, ICP and HCP limits in each species (lower limit and upper limit, Additional file 1: Fig. S5a). The three categories of promoters were determined as follows: HCPs have a CpG ratio above the upper limit and a GC content above 55%; LCPs have a CpG ratio below the lower limit and ICPs are neither HCPs nor LCPs. For promoter methylation analysis, only promoters having more than 35% of their CpGs covered in WGBS were considered. The metaplots of CG methylation in genes (Fig. 1d) were generated by calculating the average CG methylation in twenty equal-sized windows for each annotated Ensembl gene on autosomes and ten 1 kb windows of flanking sequences. The calculation of gene body methylation was performed by merging all isoforms of the same gene to create one annotation per gene, and averaging the methylation of CpGs from +500 bp after the TSS to the end of the gene annotation. For 'gg-dko' germline genes, we used the list of mouse germline genes upregulated in *Dnmt3a/b* double knockout embryos [6] and human orthologs were retrieved using Ensembl Biomart with the Ensembl 99 database and the Human genes GRCh38.p13 dataset. For the remaining species, orthologs were retrieved based on the ‘gg dko’ human orthologs. We calculated the enrichment of 'gg dko' orthologs among genes upregulated by 5-azadC using as background all genes or genes with a methylated CG-rich promoter with hypergeometric tests adjusted with Benjamini-Hochberg correction for multiple testing.

### Identification of PMDs and DMVs

MethylSeekR [20] was used to identify partially methylated domains (PMDs) and unmethylated regions (UMRs) in the different species using the parameters meth.cutoff=0.5 and nCpG.cutoff=5. DMVs were defined as UMRs with a size >= 5000 bp. We identified genes overlapping DMVs in each species and then used the human orthologs of these genes in order to compare the lists between species. The percentage of conservation between species in the Fig. 3d was calculated using the formula 100*n/min(x, y) where n is the number of common genes overlapping DMVs between two species and x and y are the numbers of genes overlapping DMVs in each species.

### Prediction of imprinted DMRs

Imprinted DMRs were identified using custom bash scripts using bedtools and awk. We used the read information from the CpG_context bismark output file and only kept reads that covered at least 3 CpGs. We devided the genomes in windows of 50 bp sliding by steps of 10 bp, and selected windows with a mean methylation between 30 and 60%, with more than 90% of their overlapping reads being either fully methylated or unmethylated and with a difference between the percentage of fully methylated and fully unmethylated reads below 40. Consecutive windows passing these cut-offs were merged. We excluded regions on chromosome X and selected regions with at least 20 CpGs and a size of 350 bp in the stringent mode and with at least 10 CpGs and no size threshold in the lenient mode. These parameters have been adjusted and optimized on the known mouse gDMRs. The bioinformatic code is provided in the Additional file 11.

### Motifs enrichment

The analysis of motif enrichment and distribution of motifs in peaks was performed using TFmotifView [57] with motifs from the JASPAR2020 database. Briefly, control regions were selected randomly within the same chromosome from regions with matched CpG content. Allelic methylation regions and shuffled control regions were searched for motif occurrences using MAST v5.1.0 (from the MEME suite) with a dynamic *p*-value threshold based on the motif information content (IC) (*p*-value = 1/2IC). The statistical significance of the motif enrichment in peaks over control regions was assessed using a hypergeometric *p*-value.

### RNA-seq library preparation

RNA-seq libraries were prepared from 150 to 350 ng of total RNA using the TruSeq Stranded Total RNA Library Prep Gold kit (Illumina), according to the manufacturer's instructions. Briefly, cytoplasmic and mitochondrial ribosomal RNA (rRNA) was removed using biotinylated, target-specific oligos combined with Ribo-Zero rRNA removal beads. The depleted RNA was fragmented using divalent cations at 94°C and cleaved RNA fragments were copied into first strand cDNA using reverse transcriptase and random primers followed by second strand cDNA synthesis using DNA Polymerase I and RNase H. cDNA fragments were blunted, adenylated and ligated to adapters using T4 DNA Ligase. The cDNA libraries were generated with 12 cycles of PCR amplification, purified using AMPure XP beads (Beckman-Coulter) and checked for quality and quantified using capillary electrophoresis. Single-end sequencing (1 × 50bp) was performed on an Illumina HiSeq 4000.

### RNA-seq analysis

Quality control checks on sequencing reads were performed with FastQC and reads were mapped to the corresponding genome with HiSat2 (v2.0.5). For data visualization, we generated BigWig files of normalized read counts per base with bamToBed from bedtools and bedGraphToBigWig from UCSC using only reads that map uniquely in the genome. We calculated raw read counts in Ensembl exons from the BAM files with HTseqcount (v0.9.1) and used these counts to identify differentially expressed genes with DESeq2 (v1.20.0). Genes were called differentially expressed if they have a fold change > 3 and an adjusted *p*-value < 0.01. Normalized counts and Reads Per Kilobase of exon per Million fragments mapped (RPKM) scores were calculated with the ‘counts’ and ‘rpkm’ functions of DESeq2. For measuring expression of transposable elements, we counted unique and multiple-mapping reads in TE families using featureCounts from the Rsubread package (v1.30.9) with the option to weight multi-mapping reads by the number of mapping sites (parameters countMultiMappingReads = TRUE, fraction = TRUE, useMetaFeatures = TRUE). Differentially expressed TE families were identified using DESeq2 (v1.20.0) with a log2 fold change > 0.5 and an adjusted *p*-value < 0.05.

### RT-qPCR

RNAs were reverse transcribed with the Maxima first strand cDNA synthesis kit (Thermo Scientific) using a combination of oligo (dT) and random hexamer primers. RT-qPCR was performed with the Kapa Mix (Clinisciences) on a StepOnePlus realtime PCR system (Life Technologies). We used fast PCR cycling conditions as follows: 95°C for 20 s, 40 cycles (95°C for 20 s, 64°C for 30 s), followed by a dissociation curve. The level of expression was normalized with two housekeeping genes (*Gusb* and *Mrpl32*). The primer sequences are available in the Table S10.

### Gene ontology

For each gene ontology biological process, we calculated the enrichment and associated hypergeometric *p*-values of genes in each class compared to all genes. *P*-values were then adjusted with Benjamini-Hochberg correction for multiple testing. Because of the lack of gene ontology annotations in rabbit, gene ontology enrichment analysis for rabbit were conducted using human orthologs.

## Supplementary Information

**Additional file 1:** **Figs. S1-S7**. **Figure S1:** Expression of *DNMT* genes and additional analysis of vertebrate methylomes in muscle and sperm. **Figure S2:** Analysis of DNA methylation in CG-rich regions in vertebrate muscle datasets. **Figure S3:** Analysis of DNA methylation in CG-rich regions of vertebrate gametes. **Figure S4:** Correlation between gene body methylation and gene expression in vertebrates. **Figure S5:** Promoter classification and analysis of promoter DNA methylation in vertebrates. **Figure S6:** Genome-wide DNA methylation and gene expression patterns upon 5azadC treatment in dermal fibroblasts. **Figure S7:** Reactivation of transposable elements by 5azadC treatment in vertebrates.

**Additional file 2: Table S1.** Summary of WGBS sequencing statistics.

**Additional file 3: Table S2.** Summary of RRBS sequencing statistics.

**Additional file 4: Table S3.** Gene ontology analysis of DMVs.

**Additional file 5: Table S4.** Table of predicted imprinted DMRs.

**Additional file 6: Table S5.** Table of enriched motifs in predicted imprinted DMRs.

**Additional file 7: Table S6.** Summary of RNA-seq sequencing statistics.

**Additional file 8: Table S7.** Gene ontology analysis of methylated CpG-rich promoters.

**Additional file 9: Table S8.** List of 'gg dko' orthologs in each species.

**Additional file 10: Table S9.** Differential gene expression analysis by RNA-seq after 5-azadC treatment.

**Additional file 11.** Bioinformatic code for the prediction of imprinted DMRs.

**Additional file 12: Table S10.** Primer sequences.

## Data Availability

The WGBS, RRBS and RNA-seq datasets generated during the study are available in the NCBI Gene Expression Omnibus (GEO) database under the accession number GSE175615 [58]. We also used the following publicly available datasets downloaded from NCBI GEO or SRA: WGBS Hsap muscle (GSM1282360) [59], WGBS Hsap sperm (GSM752295) [60], WGBS Mmus sperm (GSM1202750) [61], WGBS Btau muscle (GSM2615740) [62], WGBS Btau sperm (GSM2840125) [63], WGBS Cfam sperm (GSM2098426) [14], WGBS Sscr muscle (GSM3374919) [64], WGBS Ggal muscle (SRR5015166) [65], WGBS Ggal sperm (GSM1366300) [66], WGBS Drerio muscle (SRP020008) [67], WGBS Hsap oocyte (JGAS00000000006) [68], WGBS Mmus oocyte (GSM1386019) [69], WGBS Btau oocyte (GSM4275395) [40], RRBS Mmus sperm (GSM1471911) [8], RRBS Btau sperm (GSM2729810) [70]. The bioinformatic code for prediction of imprinted DMRs is provided in the Additional file 11.

## Abbreviations

5mC: 5-methylcytosine
CDS: Coding sequence
CGI: CpG island
DMR: Differentially methylated region
DMV: DNA methylation valley
DNMT: DNA methyltransferase
gDMR: Germline differentially methylated region
HCP: High CpG promoter
ICP: Intermediate CpG promoter
LCP: Low CpG promoter
pCGI: Promoter CpG island
RRBS: Reduced representation bisulfite sequencing
WGBS: Whole genome bisulfite sequencing
XCI: X-chromosome inactivation
