# Supplementary material for: A comparative methylome analysis reveals conservation and divergence of DNA methylation patterns and functions in vertebrates
Source: BMC Biol. 2022 Mar 23;20:70. doi: 10.1186/s12915-022-01270-x (PMC8941758; doi:10.1186/s12915-022-01270-x)
Supplement: Supplementary file 1 — Additional file 1: Figs. S1-S7. Figure S1: Expression of DNMT genes and additional analysis of vertebrate methylomes in muscle and sperm. Figure S2: Analysis of DNA methylation in CG-rich regions in vertebrate muscle datasets. Figure S3: Analysis of DNA methylation in CG-rich regions of vertebrate gametes. Figure S4: Correlation between gene body methylation and gene expression in vertebrates. Figure S5: Promoter classification and analysis of promoter DNA methylation in vertebrates. Figure S6: Genome-wide DNA methylation and gene expression patterns upon 5azadC treatment in dermal fibroblasts. Figure S7: Reactivation of transposable elements by 5azadC treatment in vertebrates. [file 12915_2022_1270_MOESM1_ESM.pdf]

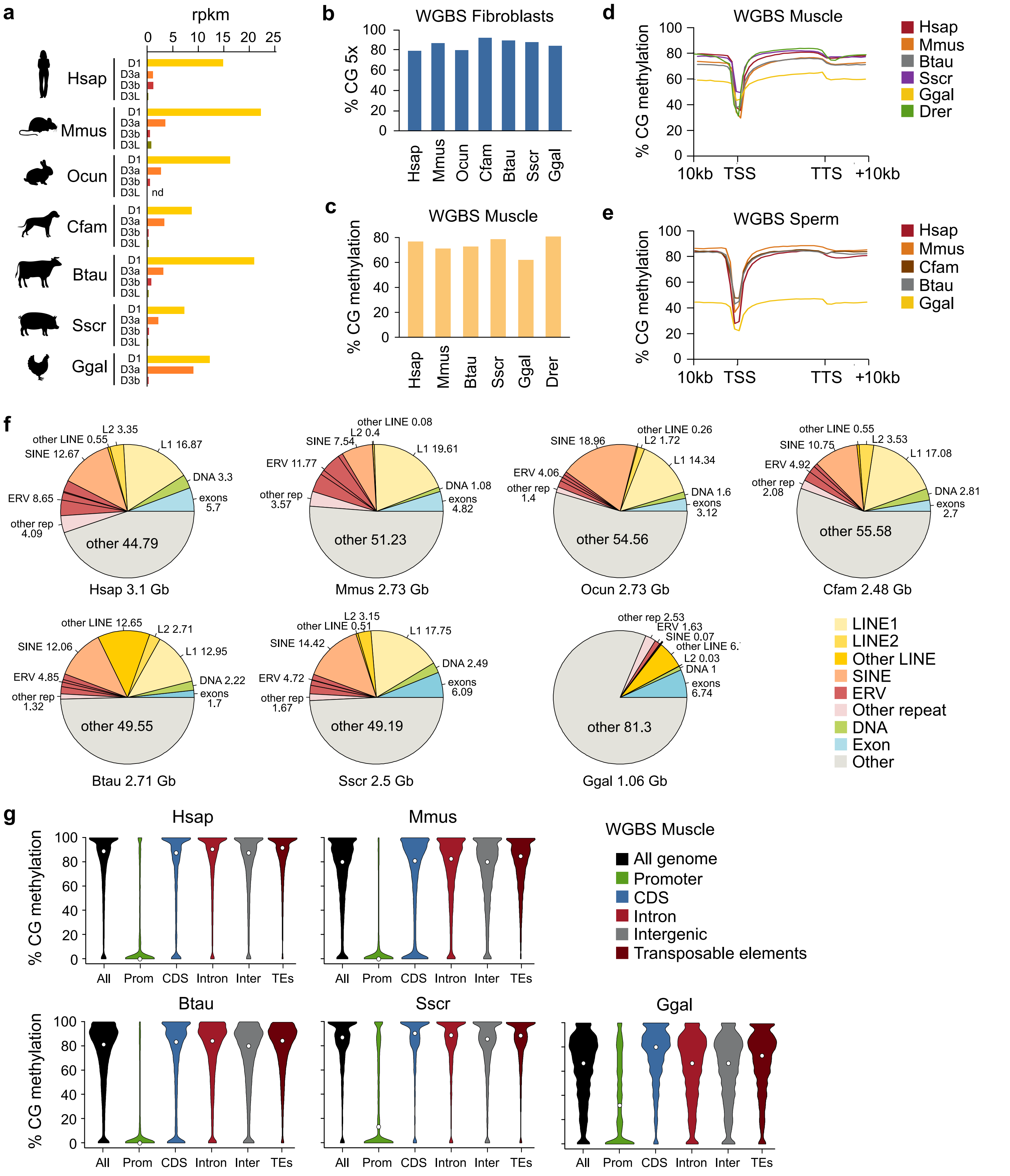

Figure S1: Expression of *Dnmt* genes and additional analysis of vertebrate methylomes in muscle and sperm. (a) Expression of *Dnmt* genes measured by RNA-seq in dermal fibroblasts from the seven studied species. nd: not determined because of the lack of *Dnmt3L* annotation in the rabbit genome. **(b)** Percentage of CGs with at least 5 unique reads in WGBS datasets from dermal fibroblasts. **(c)** Average methylation of CG sites with at least 5 unique reads in muscle WGBS datasets. **(d)** Metaplot of CG methylation levels over Ensembl genes and 10 kb flanking sequences calculated from muscle WGBS datasets. TSS: Transcription start site, TTS: Transcription termination site. **(e)** Metaplot of CG methylation levels over Ensembl genes and 10 kb flanking sequences calculated from sperm WGBS datasets. **(f)** Genome composition for the seven vertebrate species based on Ensembl and RepeatMasker annotations. **(g)** Violin plots of CG methylation levels in different genomic features calculated from muscle WGBS datasets. Median values are indicated by white circles.

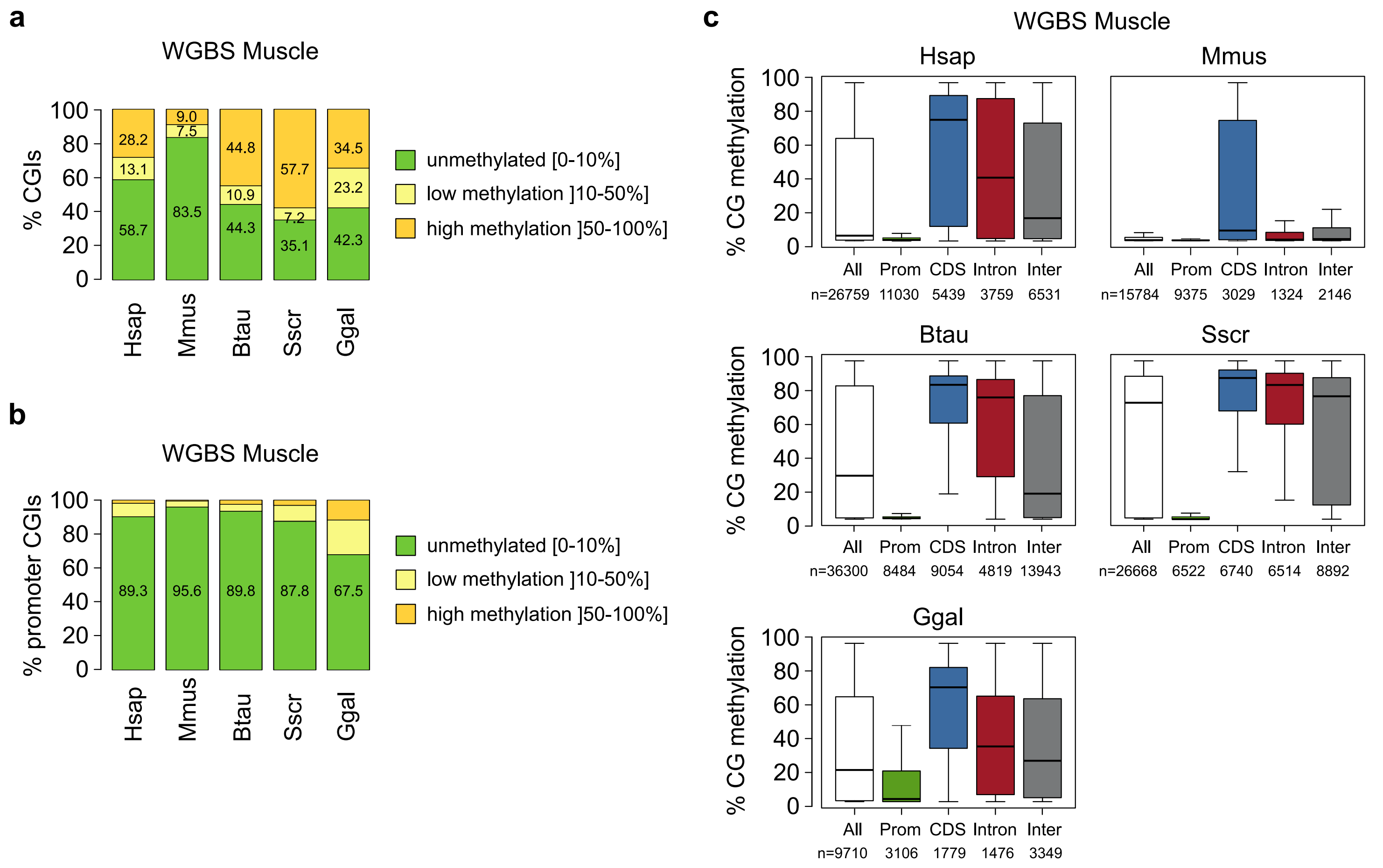

Figure S2: Analysis of DNA methylation of CG-rich regions in vertebrate muscle datasets. (a-b) Stacked bar graph representing the proportions of CGIs (a) and promoter-CGIs (b) according to their mean CG methylation in muscle WGBS datasets. The percent values are indicated on the graphs. **(c)** Box plots of the mean CG methylation of CGIs overlapping TSS (prom), coding sequences (CDS), introns or intergenic regions (inter) in muscles. The line in the boxplots indicates the median, the box limits indicate the upper and lower quartiles and the whiskers extend to 1.5 IQR from the quartiles. The numbers of CGIs in each category are indicated below the graphs.

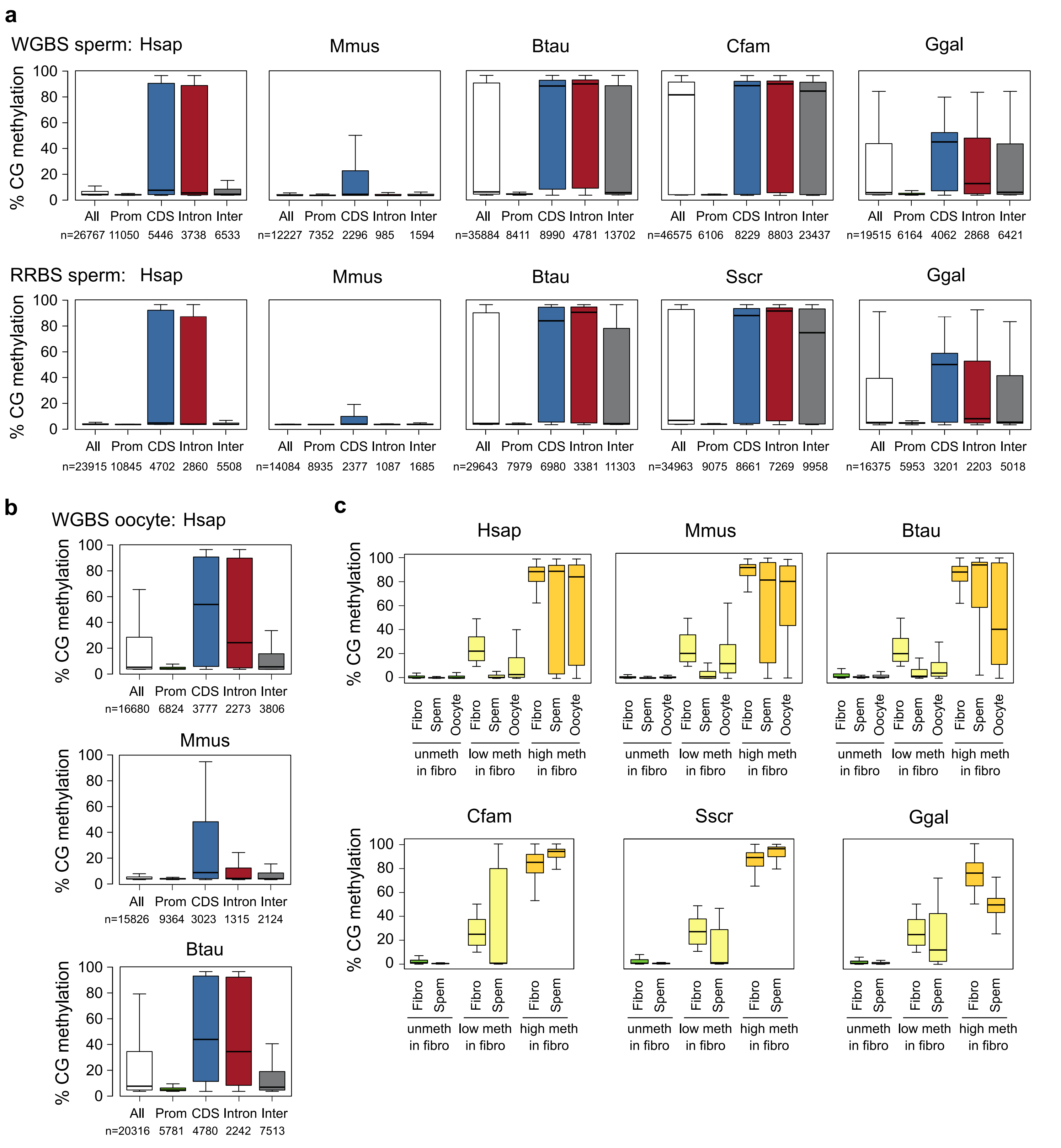

Figure S3: Analysis of DNA methylation of CG-rich regions in vertebrate gametes.(a) Box plots of the mean CG methylation of CGIs overlapping TSS (prom), coding sequences (CDS), introns or intergenic regions (inter) calculated from sperm WGBS and RRBS datasets. The numbers of CGIs in each category are indicated below the graphs. **(b)** Box plots of the mean CG methylation of CGIs calculated from oocyte WGBS datasets. **(c)** Concordance between CGI methylation in dermal fibroblasts and gametes. For each species, CGIs were categorized according to their methylation in dermal fibroblasts. The box plots show the distribution of CGI methylation in fibroblasts and gametes for CGIs unmethylated in fibroblasts (<10%, green), with low methylation in fibroblasts (10-50%, yellow) or with high methylation in fibroblasts (>50%, orange). The line in the boxplots indicates the median, the box limits indicate the upper and lower quartiles and the whiskers extend to 1.5 IQR from the quartiles.

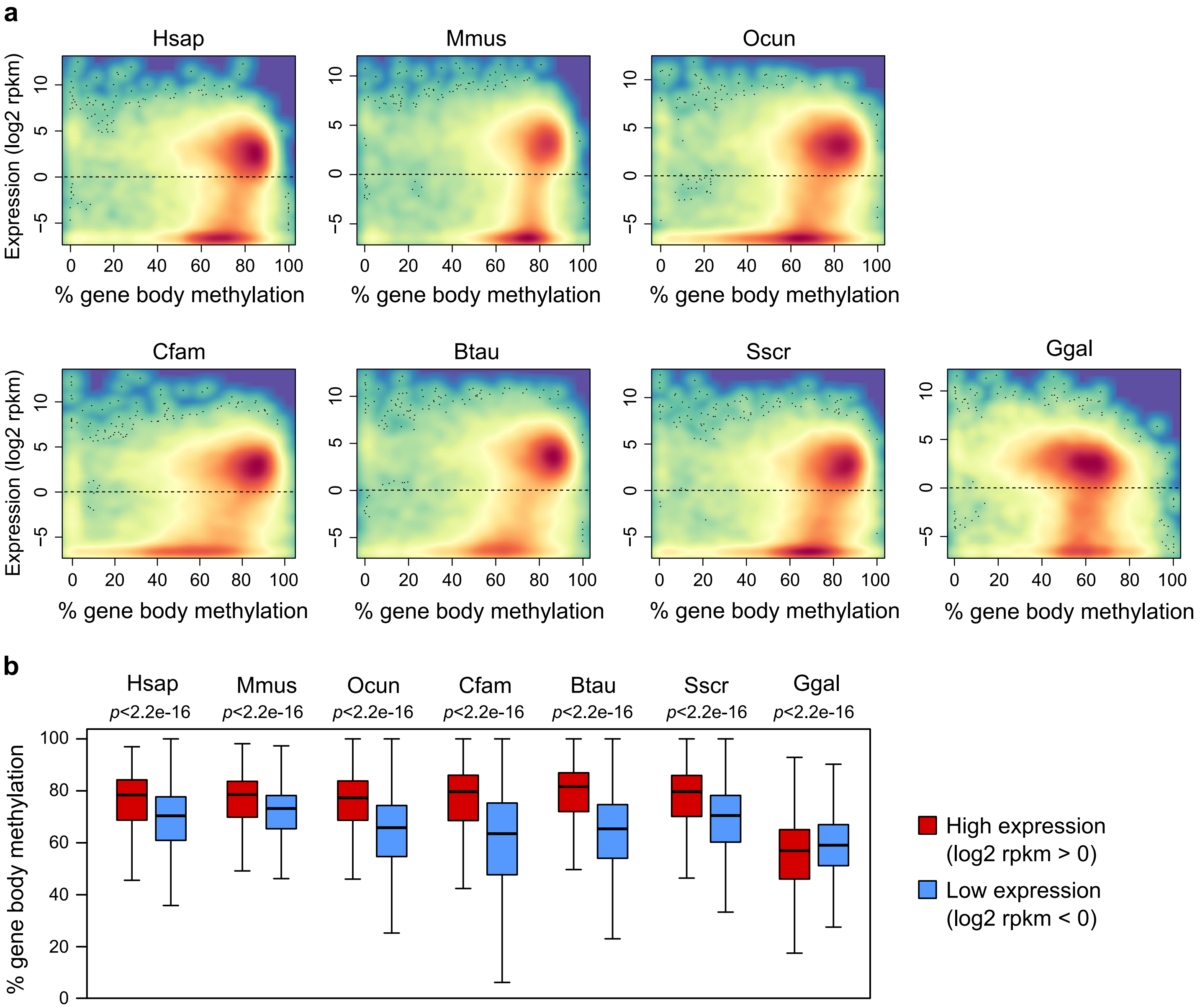

Figure S4: Correlation between gene body methylation and gene expression in vertebrates. a. Density scatter plots comparing the mean CG methylation of gene bodies (calculated by WGBS) and expression of the corresponding genes (rpkm, calculated by RNA-seq) in dermal fibroblasts from the seven studied species. The dotted lines represent the separation between genes with high (log2 rpkm > 0) and low (log2 rpkm < 0) expression. **b.** Boxplot comparing for each species the distribution of gene body methylation in genes with high expression (log2 rpkm > 0) and low expression (log2 rpkm <0). The line in the boxplots indicate the median, the box limits indicate the upper and lower quartiles and the whiskers extend to 1.5 IQR from the quartiles. *P*-values: wilcoxon test.

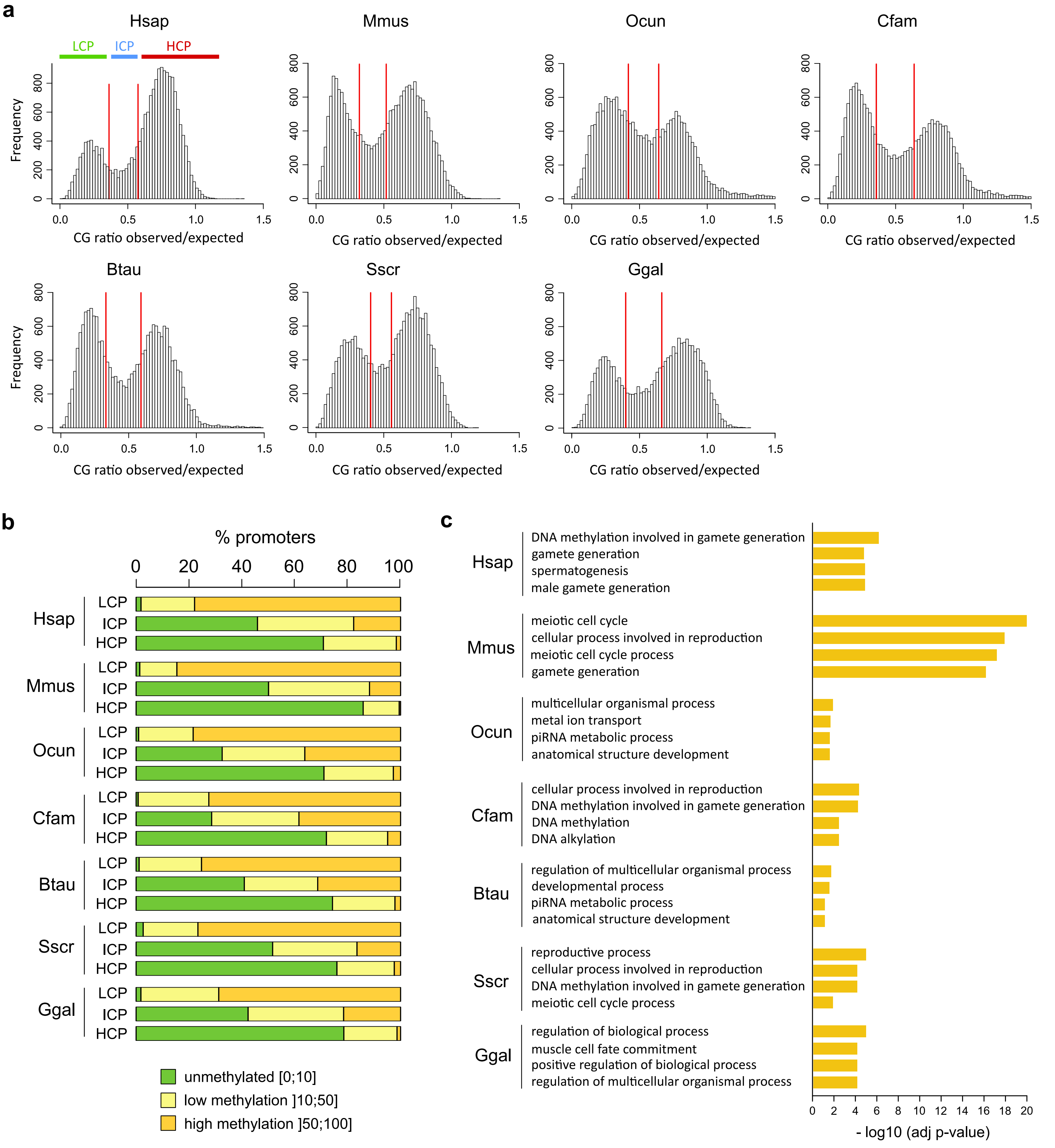

Figure S5: Promoter classification and analysis of promoter DNA methylation in vertebrates. (a) Density histograms showing the distribution of CG ratios observed/expected calculated in Ensembl gene promoters (-1000 to +500 bp from the TSS) for each species. For each species, the thresholds used to separate low (LCP), intermediate (ICP) and high CG promoters (HCP) are shown by red lines. **(b)** Stacked bar graph representing the proportions of unmethylated (<10%), lowly methylated (10-50%) and highly methylated (>50%) promoters for LCP, ICP and HCP promoter classes in dermal fibroblasts for each species. **(c)** Gene ontology enrichment analysis of highly methylated (mean methylation > 50%) CG-rich promoters (ICP and HCP). For each species, the top 4 ranked ontology terms are shown with their associated adjusted p-values (-log10, hypergeometric test).

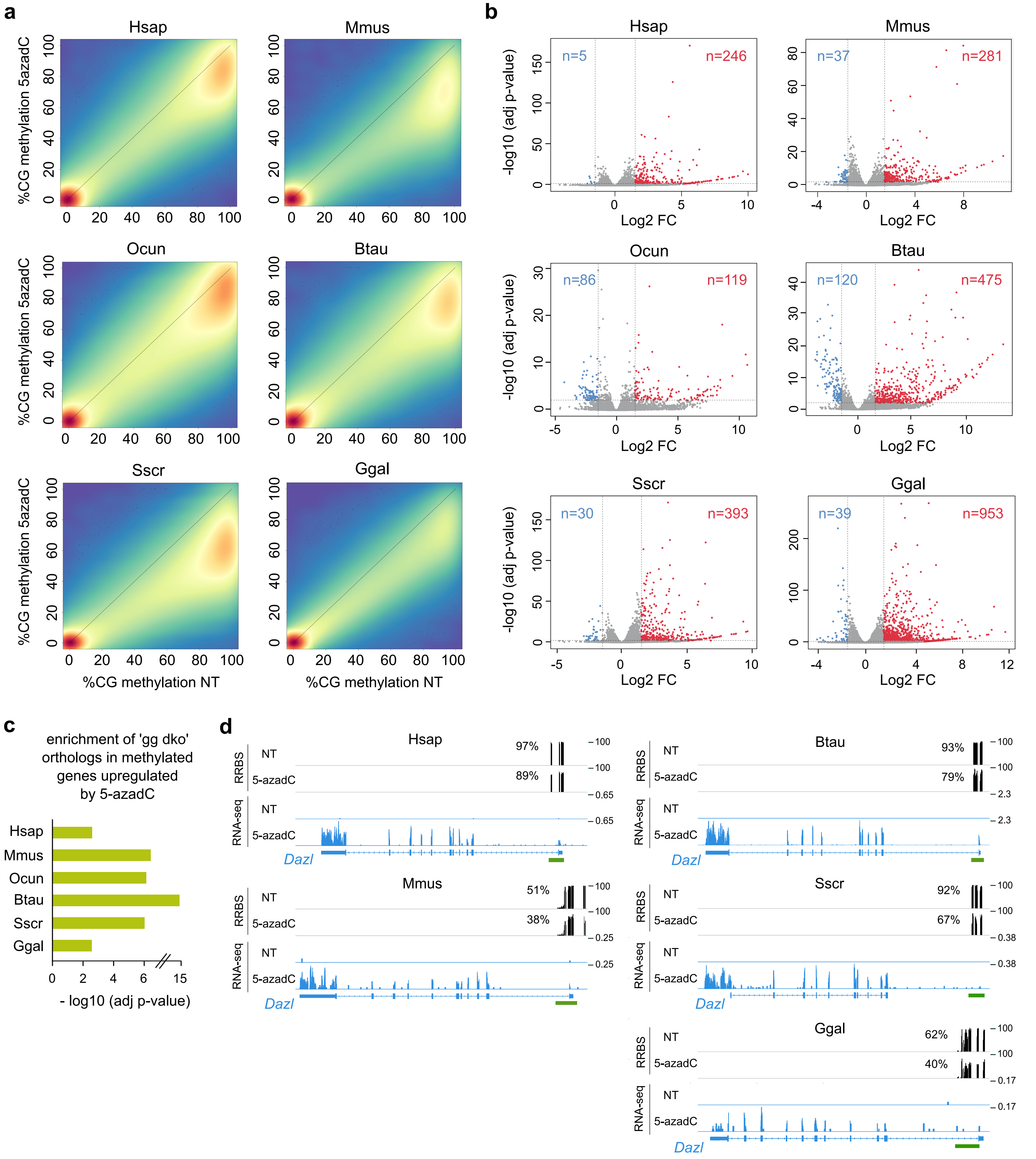

Figure S6: Genome-wide DNA methylation and gene expression patterns upon 5-azadC treatment in dermal fibroblasts. (a) Density scatter plots comparing CG methylation scores measured by RRBS in 500 bp tiles in 5-azadC treated compared to untreated (NT) dermal fibroblasts for each species. **(b)** Volcano plot representing gene expression changes measured by RNA-seq in 5-azadC treated fibroblasts compared to untreated fibroblasts in each species (n=3 independent replicates per condition). Significantly down-regulated and up-regulated genes are shown in blue and red respectively. **(c)** Enrichment of 'gg dko' orthologs among genes with a methylated CpG-rich promoter upregulated by 5-azadC in each species. The graph shows associated p-values (-log10) calculated by hypergeometric tests using all genes with a methylated CpG-rich promoter as background. **(d)** Genome browser view of RRBS and RNA-seq profiles of the *Dazl* gene in control and 5-azadC treated dermal fibroblasts of 5 species. The percentage of methylation measured by RRBS in the region covering the gene promoter is indicated. For RNA-seq, one replicate is shown per condition. CpG islands (green rectangles) and Ensembl gene annotations are shown below the tracks.

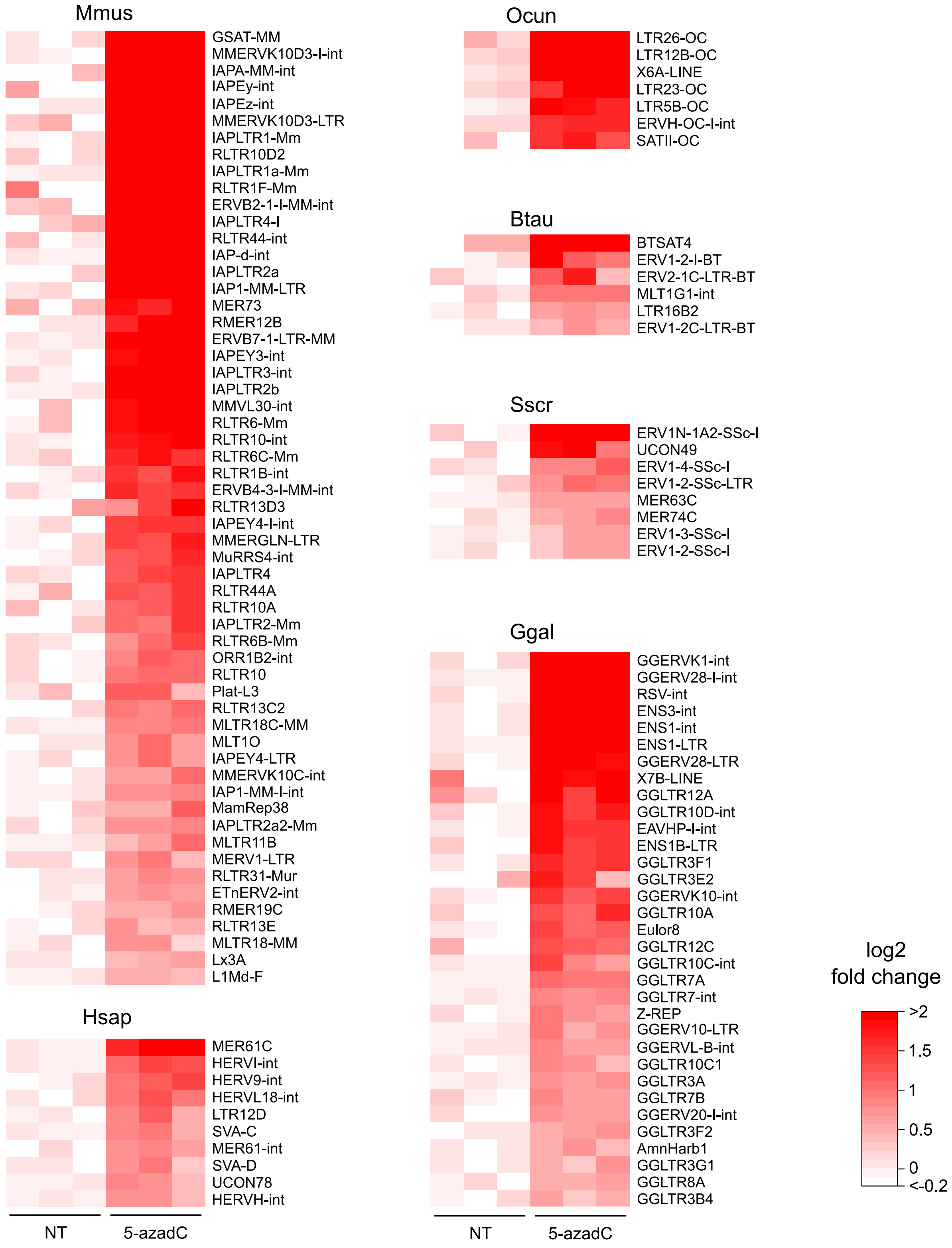

Figure S7: Reactivation of transposable elements by 5-azadC treatment in vertebrates. The heatmaps show the expression of transposable element families significantly upregulated by 5-azadC treatment in dermal fibroblasts for each species. The expression is represented as a fold change relative to the mean expression in untreated (NT) cells (n=3 independent replicates per condition).
